# 2‐Aminoethylphosphonate utilization in *Pseudomonas putida* BIRD‐1 is controlled by multiple master regulators

**DOI:** 10.1111/1462-2920.15959

**Published:** 2022-03-08

**Authors:** Andrew R. J. Murphy, David J. Scanlan, Yin Chen, Gary D. Bending, John P. Hammond, Elizabeth M. H. Wellington, Ian D. E. A. Lidbury

**Affiliations:** ^1^ School of Life Sciences University of Warwick, Gibbet Hill Road Coventry UK; ^2^ School of Agriculture, Policy, and Development University of Reading, Earley Gate, Whiteknights Reading UK; ^3^ Plants, Photosynthesis and Soil Research Cluster, School of Biosciences University of Sheffield Sheffield UK

## Abstract

Bacteria possess various regulatory mechanisms to detect and coordinate a response to elemental nutrient limitation. In pseudomonads, the two‐component system regulators CbrAB, NtrBC and PhoBR, are responsible for regulating cellular response to carbon (C), nitrogen (N) and phosphorus (P) respectively. Phosphonates are reduced organophosphorus compounds produced by a broad range of biota and typified by a direct C‐P bond. Numerous pseudomonads can use the environmentally abundant phosphonate species 2‐aminoethylphosphonate (2AEP) as a source of C, N, or P, but only PhoBR has been shown to play a role in 2AEP utilization. On the other hand, utilization of 2AEP as a C and N source is considered substrate inducible. Here, using the plant‐growth‐promoting rhizobacterium *Pseudomonas putida* BIRD‐1 we present evidence that 2AEP utilization is under dual regulation and only occurs upon depletion of C, N, or P, controlled by CbrAB, NtrBC, or PhoBR respectively. However, the presence of 2AEP was necessary for full gene expression, i.e. expression was substrate inducible. Mutation of a LysR‐type regulator, termed AepR, upstream of the 2AEP transaminase‐phosphonatase system (PhnWX), confirmed this dual regulatory mechanism. To our knowledge, this is the first study identifying coordination between global stress response and substrate‐specific regulators in phosphonate metabolism.

## Introduction

**Fig. 1:**
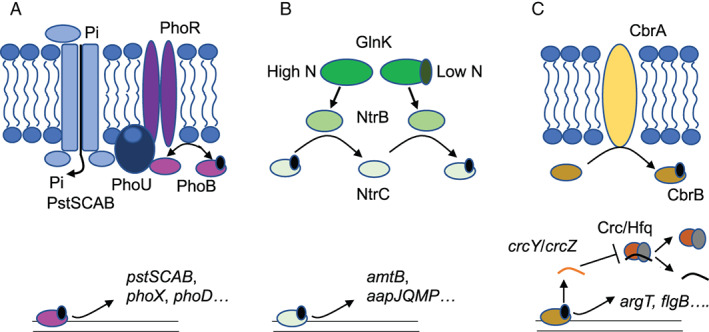
Regulators of nutrient stress in *Pseudomonas putida*. A. In response to low external Pi concentrations, the kinase activity of PhoR is de‐repressed by PstSCAB‐PhoU resulting in the phosphorylation of PhoB which then drives expression of members of the Pho regulon. B. The uridylation of GlnK in response to low cytoplasmic N concentrations activates the kinase activity of NtrB resulting in the phosphorylation of NtrC which then drives expression of members of the Ntr regulon. When cytoplasmic nitrogen concentrations are high, GlnK instead stimulates the dephosphorylase activity of NtrB. C. In response to low carbon the CbrB kinase CbrA phosphorylates CbrB, which then drives the expression of Cbr responsive genes in addition to the small RNAs *crcY* and *crcZ*. These small RNAs bind to the Crc/Hfq complex, sequestering it, and preventing it from binding targeted mRNAs and thus driving translation of Cbr responsive proteins.

The three most essential nutrients for bacterial cells, by quantity, are carbon (C), nitrogen (N) and phosphorus (P). These nutrients provide energy, building blocks for anabolism and primary and secondary metabolism, and maintain cellular homeostasis. In the environment, the ratio of these nutrients fluctuates and the concentration of any one can become limiting for cellular growth (Shimizu, 2014). To maximize resources and conserve energy bacteria possess ‘sensors’ to detect the relative depletion of a given nutrient (Groisman, 2016). These sensors can detect signals either internal or external to the cell and are usually two‐component regulatory systems, which coordinate a cellular response to nutrient depletion via the simultaneous regulation of numerous genes (regulon). Two‐component regulators typically consist of a histidine protein kinase sensor protein, which detects the stress signal, and a response regulator, which, once activated by phosphorylation by the sensor protein, acts as a transcription factor to drive transcription of its associated regulon (Zschiedrich *et al*., 2016). In pseudomonads, the two‐component regulators CbrAB (Nishijyo *et al*., 2001), NtrBC (Li and Lu, 2007) and PhoBR (Monds *et al*., 2006; Lidbury *et al*., 2016) detect and coordinate a response to C, N and P stress respectively (Fig. 1). Of these, PhoBR is widespread among bacteria (Santos‐Beneit, 2015), NtrBC is found primarily in Proteobacteria (Leigh and Dodsworth, 2007), and CbrAB has thus far been found only in the *Pseudomonadaceae* (Nishijyo *et al*., 2001; Valentini *et al*., 2014; Monteagudo‐Cascales *et al*., 2019).

Bacteria use inorganic phosphate (Pi) as their preferred source of P, and as such PhoBR coordinates the response to exogenous depletion of Pi (threshold in *Escherichia coli* = 4 μM) (Wanner, 1996). In pseudomonads, albeit with a degree of intra‐genus variation, PhoBR regulates the synthesis of multiple systems related to the efficient acquisition of organic and inorganic P compounds (Lidbury *et al*., 2016). Collectively, these include alkaline phosphatases (Monds *et al*., 2006), phosphodiesterases targeting both lipid headgroups and nucleotides (Bains *et al*., 2012; Lidbury *et al*., 2016), pathways required for phosphonate degradation (Bains *et al*., 2012; Lidbury *et al*., 2016; Lidbury *et al*., 2017), the high‐affinity phosphate ATP‐binding cassette (ABC) transporter, *pstSCAB* (Monds *et al*., 2006) and the *ptx* and *htx* operons, which transport and oxidize phosphite and hypophosphite respectively (White and Metcalf, 2004). Additionally, PhoBR is involved in pathogenicity in *Pseudomonas aeruginosa*, with defects in swarming motility and cytotoxicity observed in *phoB* and *phoR* mutants even under phosphate replete conditions (Bains *et al*., 2012). Likewise, ammonium is the preferred source of N for pseudomonads, and during times of ammonium scarcity NtrBC induces the ammonium transporter AmtB as well as expression of genes required for scavenging N from alternative N sources, such as amino acids and nitrate/nitrite (Li and Lu, 2007; Zhang and Rainey, 2008). NtrBC also controls the expression of genes involved in dinitrogen fixation in diazotrophs, e.g. the rhizobacterium *P*. *stutzeri* (Zhimin *et al*., 2021). An *ntrBC* mutant of *P*. *aeruginosa* PA14 also shows defects in both swarming motility and biofilm formation, and pathogenicity is impacted due to poor dissemination within a murine host (Alford *et al*., 2020). Pseudomonads appear to use TCA cycle intermediates as their preferred C source (Rojo, 2010), and use a catabolite repression system to control the synthesis of non‐preferential C source transporters/catabolic enzymes. This catabolite repression occurs post‐transcriptionally, using the Crc/Hfq system (Moreno *et al*., 2015). This system binds and sequesters mRNA, preventing its translation. In response to low C availability, CbrB drives the expression of *crcZ* (Sonnleitner *et al*., 2009), and in *P*. *putida* also *crcY* (Moreno *et al*., 2012; García‐Mauriño *et al*., 2013). *cbrAB* mutants of *P*. *aeruginosa* and *P*. *fluorescens* are thus unable to use some C sources, such as histidine and arginine (Li and Lu, 2007; Zhang and Rainey, 2008). PhoBR also interacts with C, N, sulfur, and iron regulatory networks to coordinate gene activation under various nutrient limiting growth conditions, across different bacteria (Santos‐Beneit, 2015). Furthermore, *cbrAB*:*ntrBC* double mutants are unable to use some N sources that *ntrBC* single mutants can, indicating the connection between these regulons (Li and Lu, 2007; Zhang and Rainey, 2008). A *cbrA* mutant also shows defects in swarming motility but enhanced biofilm formation and cytotoxicity (Yeung *et al*., 2011). Thus, PhoBR, NtrBC and CbrAB can co‐regulate the same gene sets, particularly those linked with survival through adaptation to varying environmental conditions.

Phosphonates are organophosphorus molecules that contain a direct C‐P bond (Villarreal‐Chiu *et al*., 2012). While less common than the C‐oxygen (O)‐P ester bond found in other organic P molecules, phosphonates are found in a wide range of environments including freshwater lakes (Cade‐Menun *et al*., 2006), marine systems (Clark *et al*., 1998; Kolowith *et al*., 2001; Young and Ingall, 2010) and soils (Tate and Newman, 1982; Turner *et al*., 2004). The vast majority of phosphonate biosynthesis reactions begin with the isomerisation of phosphoenolpyruvate to phosphonopyruvate via phosphoenolpyruvate mutase (PepM) (Yu *et al*., 2013). Using *pepM* as a marker gene, phosphonate biosynthesis is ubiquitous in marine, soil and gut microbiomes (Villarreal‐Chiu *et al*., 2012; Peck and van der Donk, 2013; Yu *et al*., 2013; Ju *et al*., 2014; Chin *et al*., 2016). Consequently, phosphonate catabolism genes are also prevalent in distinct environments. 2‐aminoethylphosphonate (2AEP) is considered the most abundant phosphonate in nature (White and Metcalf, 2004) although to our knowledge *in situ* analytical estimates are lacking. Our previous work identified three transporters responsible for 2AEP uptake in the soil rhizobacteria *Pseudomonas putida* BIRD‐1 (hereafter BIRD‐1) (Murphy *et al*., 2021). Two of these are ABC transporters, AepXVW and AepSTU, whilst the third, AepP, is a member of the organophosphate: phosphate antiporter subfamily of major facilitator transporters (Lemieux *et al*., 2005; Law *et al*., 2009). AepSTU was constitutively synthesized and only played a minor role in facilitating 2AEP uptake as a sole P source, whilst AepXVW was the major transporter facilitating 2AEP uptake during P limiting growth conditions but played no role in 2AEP uptake under N limitation. AepP was essential for 2AEP uptake under N limitation but could facilitate growth on 2AEP as a sole P source in the absence of a functional AepXVW and AepSTU. BIRD‐1 possesses a single phosphonate catabolism system, a two‐enzyme complex containing the 2AEP‐pyruvate transaminase (PhnW) and the phosphonoacetaldehyde hydrolase (PhnX) otherwise known as phosphonatase (Jiang *et al*., 1995; Kim *et al*., 2002), which is essential for 2AEP catabolism in BIRD‐1 (Murphy *et al*., 2021). The abundance of *aepX* and genes encoding 2AEP‐specific catabolic enzymes throughout marine systems suggests that catabolism, and thus also biosynthesis of 2AEP, occurs at a greater rate than that of other phosphonates (Murphy *et al*., 2021).

In various bacteria regulation of 2AEP transport and subsequent intracellular catabolism is under the control of PhoBR, such as the *phnCDEFGHIJKLMNOP* operon encoding C‐P lyase in *Escherichia coli* or the *phnSTU‐phnWX* operon of *Salmonella enterica* (Jiang *et al*., 1995). In other bacteria, regulation is thought to be substrate inducible or phosphate‐insensitive (Ternan and Quinn, 1998; Cooley *et al*., 2011; Chin *et al*., 2018). Substrate induction through LysR‐type regulators has been demonstrated for phosphonoacetate (PhnR) (Kulakova *et al*., 2001) and phosphonoalanine (PalR) (Kulakova *et al*., 2009). LysR‐type regulators (referred to as AepR), suggested to play an analogous role in the regulation of 2AEP degradation (Borisova *et al*., 2011), are found as part of 2AEP operons though these are not closely related to either PhnR or PalR (Murphy *et al*., 2021). An *aepR* homologue has been shown to be essential to complement an *E*. *coli* C‐P lyase mutant with *phnWX* (Martinez *et al*., 2010), suggesting a role in substrate induction. However, substrate induction does not preclude other forms of regulation, and the differential expression of the 2AEP transporters of BIRD‐1 suggests other regulatory mechanisms (Murphy *et al*., 2021). Here, we investigate the regulation of 2AEP utilization (transport and catabolism) within BIRD‐1 using a combination of bacterial genetics, proteomics and promoter reporter assays. These data reveal a dual mechanism of regulation, whereby gene expression is substrate inducible, demonstrated by mutation of *aepR* upstream of *phnWX*, but requires coordination by the master regulators CbrAB, NtrBC and PhoBR, under C, N and P limitation respectively.

## Results

### PhoBR is essential for growth on 2‐aminoethylphosphonate as a sole source of P

**Fig. 2:**
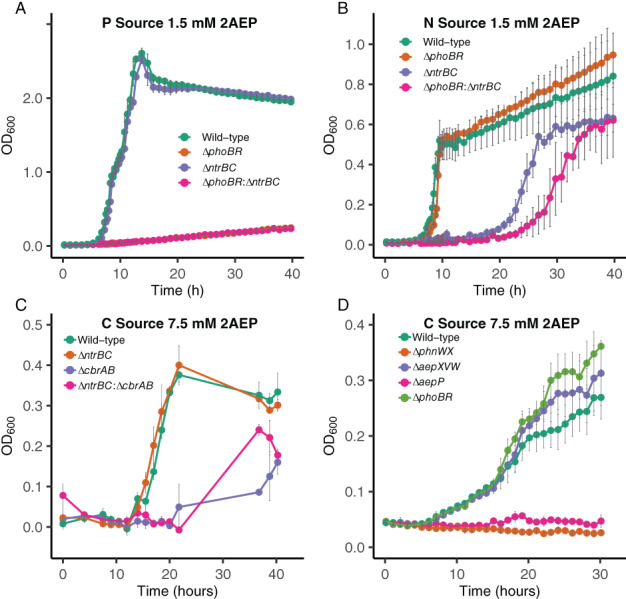
2AEP utilization by *P*. *putida* BIRD‐1 as the sole P, N or C source. Growth (*n* = 4) of *P*. *putida* BIRD‐1 wild‐type, Δ*phoBR*, Δ*ntrBC* and Δ*phoBR*:Δ*ntrBC* on 2AEP (1.5 mM) as the sole P source (A) or sole N source (B). Growth (*n* = 3) of *P*. *putida* BIRD‐1 wild‐type, Δ*ntrBC*, Δ*cbrAB* and Δ*ntrBC*:Δ*cbrAB* on 2AEP (7.5 mM) as the sole C source (C). Growth (*n* = 4) of *P*. *putida* BIRD‐1 wild‐type, Δ*phnWX*, Δ*aepXVW*, Δ*aepP* and Δ*phoBR* on 2AEP (7.5 mM) as the sole C source (D). Error bars denote the standard deviation of the mean. Growth experiments were performed in 96‐well plates, with the exception of the data presented in panel (C), which were performed in 25 ml universals containing a volume of 5 ml.

We have previously shown that BIRD‐1 synthesizes distinct transport systems to facilitate growth on 2AEP as either the sole N or P source (Murphy *et al*., 2021). In response to Pi‐limitation, BIRD‐1 synthesizes AepXVW (2AEP transporter) and PhnWX (the 2AEP‐pyruvate transaminase‐phosphonatase system) in a PhoBR‐dependent manner (Lidbury *et al*., 2016). This suggested a role for master regulators in the utilization of 2AEP as the sole P source, and potentially, N and C sources. Mutation of *phoBR* eliminated growth of BIRD‐1 on 2AEP as the sole P source (Fig. 2A), suggesting that *phoBR* is essential for 2AEP utilization when used as a sole P source. Mutation of *phoBR* had no effect on growth when 2AEP was the sole N source (Fig. 2B). This is consistent with the synthesis of another 2AEP transporter (AepP) during growth on 2AEP as the sole N source (Murphy *et al*., 2021) and suggests PhnWX is also regulated by the N‐stress response regulator NtrBC. Taken together, this suggests PhoBR is essential for 2AEP utilization as the sole P source by tightly controlling the synthesis of PhnWX, despite the fact that a basal level of AepP is expressed in this condition (Murphy *et al*., 2021).

### NtrBC and CbrAB are involved in the utilization of 2AEP as either a sole N or sole C source

Given that mutation of *phoBR* did not affect growth on 2AEP as the sole N source, we predicted that the master regulator of the N‐stress response, NtrBC, may also be involved. Generation of a Δ*ntrBC* mutant revealed NtrBC was required for efficient 2AEP utilization (Fig. 2B) but did not affect 2AEP utilization as the sole P (Fig. 2A) or sole C source (Fig. 2C). Generation of a Δ*phoBR*:Δ*ntrBC* double master regulator mutant further delayed growth on 2AEP as the sole N source (Fig. 2B) and again eliminated growth on 2AEP as the sole P source (Fig. 2A). Mutation of the genes encoding either the 2AEP antiporter (*aepP*) or phosphonatase (*phnWX*) in the Δ*ntrBC* background (Δ*ntrBC*:Δ*aepP* and Δ*ntrBC*:Δ*phnWX*) resulted in complete elimination of growth on 2AEP as the sole N source, suggesting 2AEP partially induces both AepP and PhnWX synthesis in the Δ*ntrBC* mutant at a level sufficient to support partial growth (Fig. S1).

Wild‐type Δ*phoBR* and Δ*ntrBC* were able to grow on 2AEP as the sole C source (Fig. 2C and D). Therefore, we predicted that the C stress‐response master regulator CbrAB also interacted with AepP and PhnWX. Indeed, mutation of *cbrAB* in both the wild‐type background (Δ*cbrAB*) and the *ntrBC* mutant (Δ*ntrBC*:Δ*cbrAB*) severely inhibited growth on 2AEP as the sole C source at comparable amounts (Fig. 2C). Δ*cbrAB* showed no defect in growth on 2AEP as the sole N or P source (Fig. S2). We also confirmed AepP, and not AepXVW, is the sole transporter responsible for 2AEP utilization as the sole C source (Fig. 2D) as well as an N source (Murphy *et al*., 2021). However, given poor growth still occurs despite mutation of *cbrAB*, both AepP and PhnWX must still be synthesized, as they are essential for growth on 2AEP as the sole C source (Fig. 2D), again suggesting 2AEP partially induces both AepP and PhnWX synthesis in Δ*cbrAB*, similar to induction in Δ*ntrBC* (Fig. S1). Collectively, these data suggest that both NtrBC and CbrAB likely interact with the *aepP* and/or the *phnWX* promoters, directly or indirectly. However, whilst this interaction is not essential for growth on 2AEP as either the sole N or sole C source it is necessary for efficient growth on 2AEP under these conditions.

### N limitation alone does not induce synthesis of 2AEP utilization proteins

**Fig. 3:**
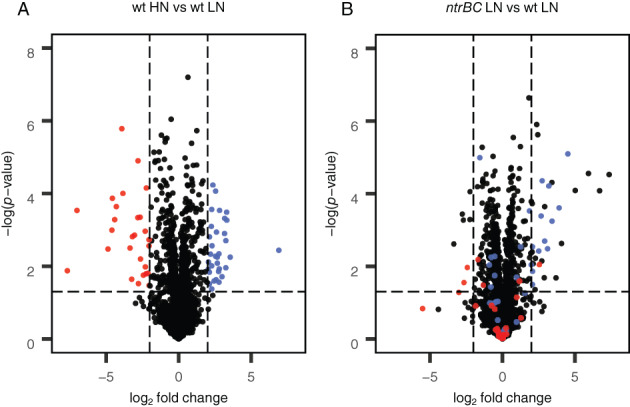
Whole‐cell protein profiles (*n* = 3) for *P*. *putida* BIRD‐1 grown under either High N (HN – 5.6 mM NH_4_) or Low N (LN – 1.5 mM NH_4_) conditions. Wild‐type HN versus wild‐type LN (A), Δ*ntrBC* LN versus wild‐type LN (B). Log_2_ fold changes represent the difference in mean Log_2_ LFQ values between each treatment. The statistical value on the *y*‐axis is generated from *Q* values (FDR corrected *p*‐values). Vertical dashed lines represent a Log_2_ LFQ difference >−2 or <2. The horizontal dashed line illustrates a cut‐off for a significant −Log_10_(*p*‐value) (*p* < 0.05). Proteins significantly upregulated in wild‐type LN versus wild‐type HN (blue) and proteins significantly downregulated in wild‐type LN versus wild‐type HN (red) are coloured in both plots.

To determine if AepP and PhnWX are synthesized in response to N limitation in the absence of 2AEP, as observed under P limitation (Lidbury *et al*., 2016), we performed comparative proteomics. Total protein was extracted from BIRD‐1 wild‐type and Δ*ntrBC* cells grown overnight on 5.6 mM NH_4_^+^ (High N) or 1.5 mM NH_4_^+^ (Low N) (Fig. 3). In total, across all strains and conditions, 1421 proteins were identified of which 60 were differentially synthesized (FDR corrected *p* < 0.05, log_2_ fold change >2, Tables S2–S4) between wild‐type High and Low N (Table 1). Thirty‐three of these proteins were significantly enriched (*p* < 0.05) in wild‐type Low N compared to High N. Comparison between wild‐type and mutant Low N proteomes suggested 16 of these were induced by NtrBC, whilst the others responded to N limitation independently of this master regulator. The putative Ntr regulon was found to include several ABC transport and catabolism proteins for various organic nitrogen compounds, including amino acids, ethanolamine, glycine betaine and urea (Table 1). Furthermore, the synthesis of several proteins linked to N metabolism was significantly downregulated in Δ*ntrBC* irrespective of nitrogen status (Fig. S3, Table 1). Whilst low level PhoBR‐dependent synthesis of AepXVW and PhnWX occurred in BIRD‐1 in response to P limitation (Lidbury *et al*., 2016), neither were detected in the wild‐type (or *ntrBC* mutant) proteome during N‐limitation. This is despite the fact NtrBC is required for efficient growth on 2AEP as the sole N source (Fig. 2B).

**Table 1:** Proteins whose synthesis is significantly upregulated in wild‐type Low N, but not Δ*ntrBC* Low N, relative to wild‐type High N, and proteins whose synthesis is significantly downregulated in both Δ*ntrBC* High and Low N, relative to wild‐type High N.

Locus tag	Gene annotation	Status	Log_2_ – Log_2_ difference (mean values)
Wild‐type Low N: wild‐type High N			
PPUBIRD1_4504	Putative IcmE/DotG Type IV secretion system protein	NtrBC dependent, Low N upregulated	3.25
PPUBIRD1_3326	CsuC Pili usher protein	NtrBC dependent, Low N upregulated	3.16
PPUBIRD1_4260	AapP Amino acid ABC transporter, ATP‐binding subunit	NtrBC dependent, Low N upregulated	2.99
PPUBIRD1_4262	AapQ Amino acid ABC transporter permease	NtrBC dependent, Low N upregulated	2.82
PPUBIRD1_1576	XdhB Xanthine dehydrogenase	NtrBC dependent, Low N upregulated	2.78
PPUBIRD1_2330	Quinohaemoprotein amine dehydrogenase A	NtrBC dependent, Low N upregulated	2.55
PPUBIRD1_0593	Ethanolamine transporter	NtrBC dependent, Low N upregulated	2.36
PPUBIRD1_1769	GlgX glycogen debranching enzyme	NtrBC dependent, Low N upregulated	2.78
PPUBIRD1_0323	Glycine betaine/l‐proline ABC transporter, ATP‐binding subunit	NtrBC dependent, Low N upregulated	2.58
PPUBIRD1_1785	NCS1 nucleoside transporter	NtrBC dependent, Low N upregulated	2.44
PPUBIRD1_1182	LivG Leucine/isoleucine/valine ABC transporter, solute‐binding protein	NtrBC dependent, Low N upregulated	2.35
PPUBIRD1_3558	NasS Nitrate‐binding protein, two‐component regulator	NtrBC dependent, Low N upregulated	2.28
PPUBIRD1_3240	Hypothetical protein	NtrBC dependent, Low N upregulated	2.28
PPUBIRD1_1577	XdhA Xanthine dehydrogenase	NtrBC dependent, Low N upregulated	2.12
PPUBIRD1_0134	Cox3 Cytochrome *c* oxidase	NtrBC dependent, Low N upregulated	2.12
PPUBIRD1_4631	Urea/BCAA ABC transporter, ATP‐binding subunit	NtrBC dependent, Low N upregulated	2.12
PPUBIRD1_4709	ThiC Thiamine biosynthesis protein	NtrBC dependent, Low N upregulated	2.05
Δ*ntrBC* High and Low N: wild‐type High N			
PPUBIRD1_2985	Hypothetical protein	NtrBC High and Low N downregulated	4.84, 4.4
PPUBIRD1_4628	Urea/BCAA ABC transporter, substrate‐binding subunit	NtrBC High and Low N downregulated	6.1, 7.13
PPUBIRD1_4838	NtrC Nitrogen stress response regulator	NtrBC High and Low N downregulated	6.56, 5.80
PPUBIRD1_5028	AmtB Ammonium channel	NtrBC High and Low N downregulated	6.25, 6.91
PPUBIRD1_4837	NtrB Nitrogen stress sensor	NtrBC High and Low N downregulated	3.79, 2.99
PPUBIRD1_2889	UreC Urease subunit alpha	NtrBC High and Low N downregulated	4.31, 4.09
PPUBIRD1_1683	Hypothetical protein	NtrBC High and Low N downregulated	3.86, 3.18

Values shown are the difference between mean Log_2_ LFQ values. Statistical significance was determined using an FDR adjusted *t*‐test, with a mean Log_2_ – Log_2_ difference threshold of 2 and an FDR of 0.05.

### 2AEP is necessary for full transcriptional activation of transport and catabolic genes

So far, our data suggested that a nutrient limitation response mediated through master regulators is required for efficient 2AEP catabolism. To investigate if this was the only mechanism of transcriptional regulation the promoter regions of both 2AEP transporters (AepXVW and AepP) and the phosphonatase pathway (PhnWX) were cloned into the *lacZ* fusion plasmid pBIO1878 (Todd *et al*., 2012; Lidbury *et al*., 2014). Three reporter plasmids, pBIO‐*aepXVW*‐pr, pBIO‐*aepP*‐pr and pBIO‐*phnWX*‐pr were transformed into BIRD‐1 wild‐type and Δ*phoBR*, Δ*ntrBC* and Δ*cbrAB* mutants. β‐galactosidase assays were performed using crude cell extracts with ortho‐nitrophenyl‐β‐galactopyranoside as the substrate. As some strains were incapable of growth on 2AEP, cultures were grown in replete media prior to washing and re‐suspension in fresh media and subsequently incubated in the various nutrient conditions for 5 h prior to assaying.

**Fig. 4:**
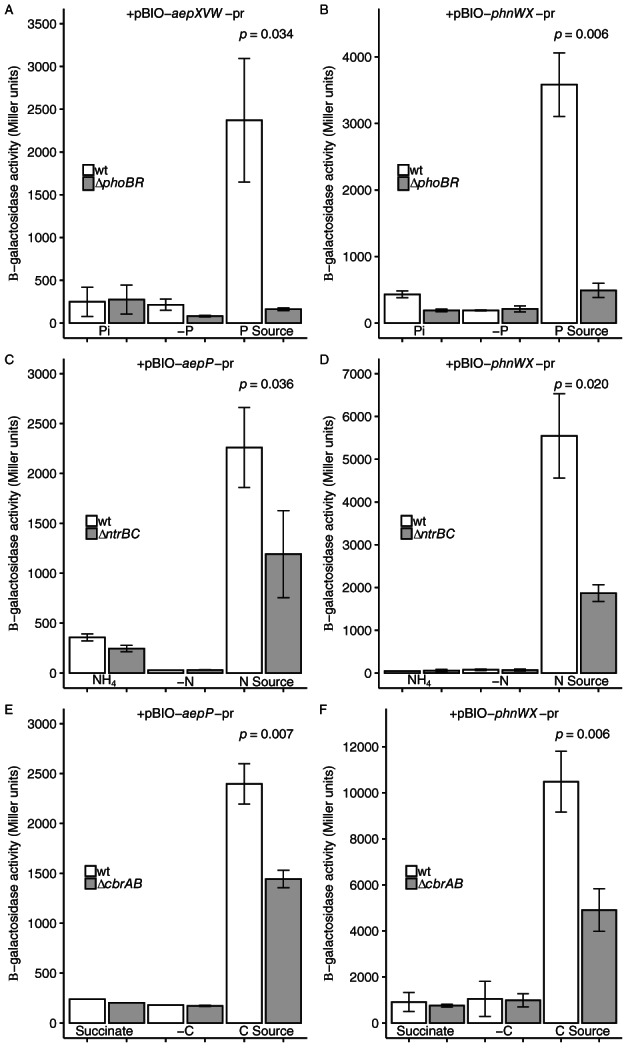
Promoter reporter assays for 2AEP‐utilization operons in *P*. *putida* BIRD‐1. β‐galactosidase activity (*n* = 3) was driven from the *aepXVW* promoter (pBIO‐*aepXVW*‐pr) (A) or the *phnWX* promoter (pBIO‐*phnWX*‐pr) (B). Wild‐type and Δ*phoBR* backgrounds are shown, under nutrient replete (Pi), deplete (‐P), or 2AEP as sole P source (P Source) conditions. β‐galactosidase activity (*n* = 3) driven from the *aepP* promoter (pBIO‐ *aepP*‐pr) (C), and the *phnWX* promoter (pBIO‐*phnWX*‐pr) (D), in wild‐type and Δ*ntrBC* backgrounds, under nutrient replete (NH_4_), deplete (‐N), 2AEP as sole N source (N Source) conditions. β‐galactosidase activity (*n* = 3) driven from the *aepP* promoter (pBIO‐*aepP*‐pr) (E) and the *phnWX* promoter (pBIO‐*phnWX*‐pr) (F), in wild‐type and Δ*cbrAB* backgrounds, under nutrient replete (succinate), deplete (‐C), 2AEP as sole C source (C Source) conditions. 2AEP was used at a concentration of 1.5 mM for all β‐galactosidase experiments. Error bars denote the standard deviation of the mean.

Despite the role of each master regulator in 2AEP utilization, β‐galactosidase assays clearly revealed that the presence of 2AEP was required for promoter activation in all our various reporter strains (Fig. 4). For pBIO‐*aepXVW*‐pr and pBIO‐*phnWX*‐pr a functional PhoBR was essential for full activation of β‐galactosidase activity when 2AEP was used as the sole P source (Fig. 4A and B). In contrast, while for pBIO‐*aepP*‐pr and pBIO‐*phnWX*‐pr a functional NtrBC was essential for full activation of β‐galactosidase activity when 2AEP was used as the sole N source (Fig. 4C and D), induction still occurred in the Δ*ntrBC* mutant. Similarly, for pBIO‐*aepP*‐pr and pBIO‐*phnWX*‐pr a functional CbrAB was essential for full activation of β‐galactosidase activity when 2AEP was the sole C source (Fig. 4E and F), induction still occurred in the Δ*cbrAB* mutant. Thus, complete expression from all three promoter regions requires substrate induction in the presence of 2AEP, and nutrient stress alone is not sufficient.

### 2AEP does not induce full expression from aminoethylphosphonate operons

**Fig. 5:**
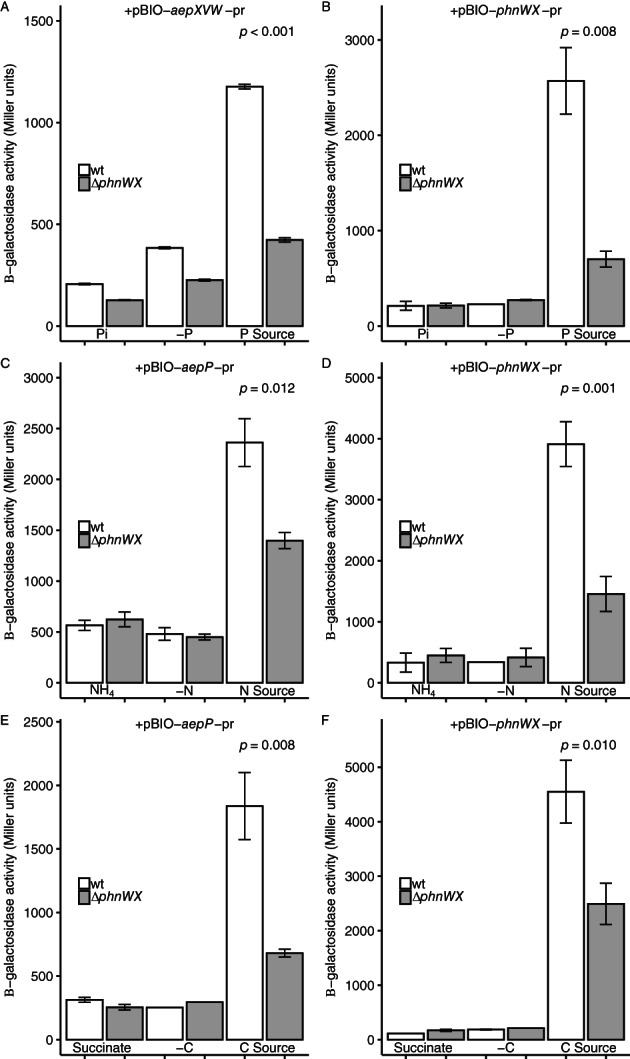
Promoter reporter assays for 2AEP‐utilization operons in the *P*. *putida* BIRD‐1 wild‐type or Δ*phnWX* mutant. β‐galactosidase activity (*n* = 3) was driven from the *aepXVW* promoter (pBIO‐*aepXVW*‐pr) (A) or the *phnWX* promoter (B). Wild‐type and Δ*phnWX* backgrounds are shown, under nutrient replete (Pi), deplete (‐P), or 2AEP as sole P source (P Source) conditions. β‐galactosidase activity (*n* = 3) driven from the *aepP* promoter (pBIO‐*aepP*‐pr) (C), and the *phnWX* promoter (pBIO‐*phnWX*‐pr) (D), in wild‐type and Δ*phnWX* backgrounds, under nutrient replete (NH_4_), deplete (‐N), 2AEP as sole N source (N Source) conditions. β‐galactosidase activity (*n* = 3) driven from the *aepP* promoter (pBIO‐*aepP*‐pr) (E) and the *phnWX* promoter (pBIO‐*phnWX*‐pr) (F), in wild‐type and Δ*phnWX* backgrounds, under nutrient replete (succinate), deplete (‐C), 2AEP as sole C source (C Source) conditions. 2AEP was used at a concentration of 1. 5 mM for all β‐galactosidase experiments. Error bars denote the standard deviation of the mean.

To determine whether 2AEP itself or a metabolite of 2AEP catabolism is responsible for activating the three aminoethylphosphonate operons, the reporter plasmids pBIO‐*aepXVW*‐pr, pBIO‐*aepP*‐pr and pBIO‐*phnWX*‐pr were transformed into BIRD‐1 Δ*phnWX*, which is incapable of growth on 2AEP as either the sole P, N (Murphy *et al*., 2021), or C source (Fig. 2D). However, as the Δ*phnWX* mutant possesses intact 2AEP transporters import of 2AEP should still happen. Relative to the wild‐type, induction by 2AEP from all aminoethylphosphonate operons was significantly reduced, but still occurred in the Δ*phnWX* mutant (Fig. 5). As such, it is likely that 2AEP, as well as an, as yet, unidentified compound contributes towards the induction of these three operons (i.e. *aepXVW*, *aepP*, *phnWX*).

### AepR^WX^ is required for efficient growth on 2AEP and substrate‐induction from the *phnWX* promoter

**Fig. 6:**
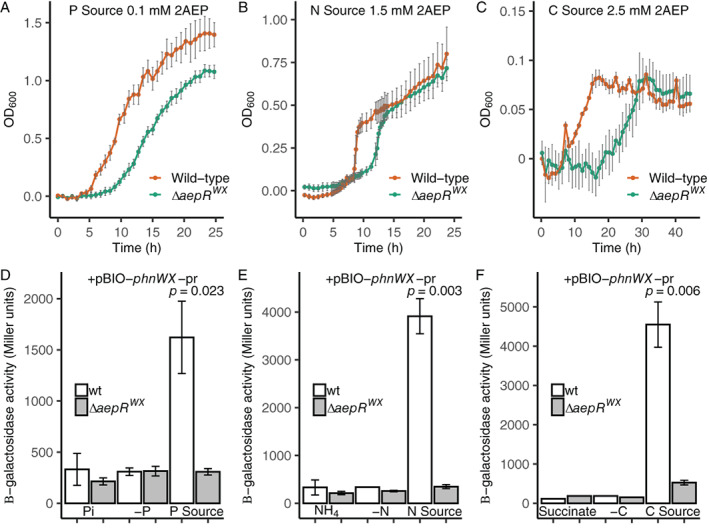
Growth and promoter reporter assays in *P*. *putida* BIRD‐1 Δ*aepR* ^*WX*^ in response to 2AEP as the sole P, N, or C source. Growth (*n* = 4) of *P*. *putida* BIRD‐1 wild‐type and Δ*aepR* ^*WX*^ on 2AEP as the sole P source (0.1 mM) (A), sole N source (1.5 mM) (B), or sole C source (2.5 mM) (C). Error bars denote the standard deviation of the mean. β‐galactosidase activity (*n* = 3) driven from the *phnWX* promoter (pBIO‐*phnWX*‐pr) in wild‐type and Δ*aepR* ^*WX*^ backgrounds, under nutrient replete (Pi), deplete (‐P), 2AEP as sole P source (P Source) (D), nutrient replete (NH_4_), deplete (‐N), 2AEP as sole N source (N Source) (E), or nutrient replete (succinate), deplete (‐C), 2AEP as sole C source (C Source) (F) conditions. Growth experiments were performed in 96‐well plates. 2AEP was used at a concentration of 1.5 mM for all β‐galactosidase experiments. Error bars denote the standard deviation of the mean.

To investigate whether the AepR proteins, encoded next to aminoethylphosphonate operons in BIRD‐1 and other organisms (Murphy *et al*., 2021), are responsible for full activation of 2AEP‐utilization genes, a mutant of the *aepR* encoded next to *phnWX* (hereafter Δ*aepR* ^*WX*^) was constructed. This *aepR* homologue was chosen as *phnWX* is essential for growth on 2AEP as the sole P, N, or C source, and as such Δ*aepR* ^*WX*^ can provide insight into all three growth conditions. Whilst growth on 2AEP as the sole P source at 1.5 mM showed no difference between wild‐type and Δ*aepR* ^*WX*^ (Fig. S4), Δ*aepR* ^*WX*^ showed slower growth compared to the wild‐type on 2AEP as the sole P source at 0.1 mM (Fig. 6A). When grown on 2AEP as the sole N (1.5 mM) or C source (2.5 mM), Δ*aepR* ^*WX*^ again showed a growth defect relative to that of the wild‐type (Fig. 6B and C). Collectively, this suggests that AepR^WX^ is involved in, but not essential for, the expression of *phnWX* when 2AEP is the sole P, N or C source.

To determine if this growth defect was due to the role of AepR^WX^ in substrate inducible activation of *phnWX*, we transformed Δ*aepR* ^*WX*^ with the reporter plasmid pBIO‐*phnWX*‐pr. In this mutant, induction of β‐galactosidase activity from pBIO‐*phnWX*‐pr was significantly impaired in comparison with wild‐type cells when 2AEP was the sole P, N, or C source (Fig. 6D–F). In fact, only Δ*aepR* ^*WX*^ cells incubated with 2AEP as a C source produced β‐galactosidase activity above the control reading (Fig. 6F). In summary, AepR^WX^ is required for full (substrate‐inducible) activation of *phnWX* and subsequent growth on 2AEP as the sole P, N, or C source, but is not essential.

## Discussion

The regulation of 2AEP uptake and catabolism has previously been considered to be either under the control of PhoBR, the master regulator of P stress (Villarreal‐Chiu *et al*., 2012; Lidbury *et al*., 2016), or otherwise substrate inducible (Ternan and Quinn, 1998). Here, we present evidence that, in BIRD‐1, two other master regulators, NtrBC and CbrAB, which coordinate the response to N and C stress respectively, are also involved in the regulation of 2AEP uptake and catabolism, as their absence impairs growth on 2AEP as their corresponding nutrient source. Previous evidence of PhoBR‐independent 2AEP catabolism in other *Pseudomonas* spp. does not rule out a role for these master regulators (Ternan and Quinn, 1998). We saw no evidence of interaction between these master regulators in the use of 2AEP as the sole P source, where PhoBR alone was essential. Neither was there any evidence of interaction between NtrBC and CbrAB in the use of 2AEP as the sole N or C source, demonstrated by the double mutants showing no differences in phenotype to the respective single master regulator mutants, unlike utilization of some other N and C containing compounds (Li and Lu, 2007; Zhang and Rainey, 2008). However, we cannot rule out an interaction between PhoBR and NtrBC/CbrAB in the use of 2AEP as the sole N or C source respectively. On the other hand, a role for such master regulators in 2AEP metabolism cannot be assumed in other bacteria, such as marine *Alphaproteobacteria*. Indeed, there is evidence consistent with substrate‐inducible regulation in both laboratory‐cultivated (Cooley *et al*., 2011; Chin *et al*., 2018) and environmental marine bacteria independent of nutrient concentration (Murphy *et al*., 2021). However, the exact mechanism or mechanisms of regulation in these organisms remains to be determined. Additionally, we showed that in BIRD‐1 transcriptional activation of the *aepP*, *aepXVW* and *phnWX* promoters was substrate‐inducible in that nutrient stress alone did not drive measurable enzyme activity in the BIRD‐1 reporter strains. Nevertheless, our data clearly identify an interaction between the nutrient stress response regulators and substrate‐inducible production of PhnWX and 2AEP transporters.

**Fig. 7:**
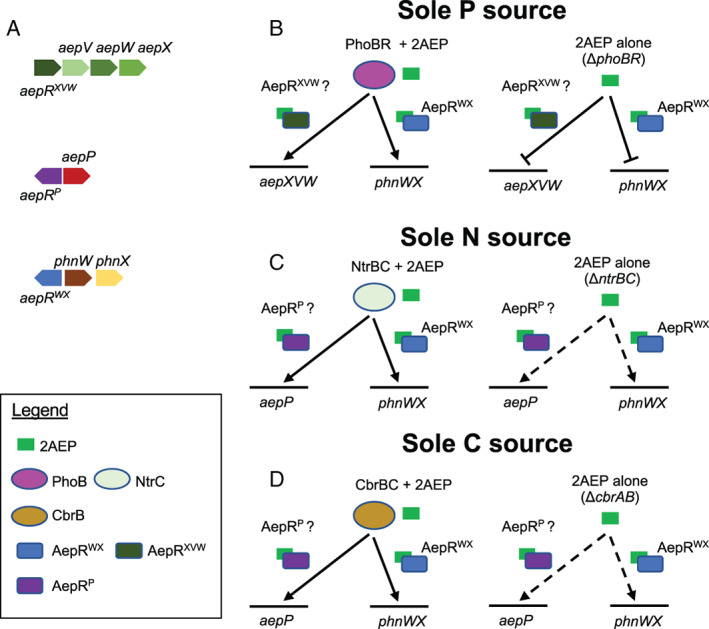
A proposed model of the interactions between the master regulators, PhoBR, NtrBC and CbrAB, and AepR regulators of *Pseudomonas putida* BIRD‐1 with 2AEP as the sole nutrient source and expression from aminoethylphosphonate promoters. Conditions under which full expression occurs are shown with filled line arrows, conditions under which partial expression occurs are shown with dashed line arrows, conditions under which no expression occurs are shown with blunt‐ended arrows. A. The aminoethylphosphonate operons of BIRD‐1. B. PhoBR together with 2AEP is required for expression from the *phnWX* and *aepXVW* promoters. Substrate induction occurs via AepR^WX^ at the *phnWX* promoter, and likely through the homologous AepR^XVW^ at the *aepXVW* promoter. In the absence of PhoBR, substrate‐induction is not sufficient for expression at either promoter, and no growth occurs. Substrate induction via AepR^WX^ at the *phnWX* promoter is not essential for growth but is required for efficient utilization of 2AEP. C. NtrBC together with 2AEP is required for complete expression from the *aepP* and *phnWX* promoters. Substrate induction occurs via AepR^WX^ at the *phnWX* promoter, and likely through the homologous AepR^P^ at the *aepP* promoter. In the absence of NtrBC, substrate induction drives expression at both promoters, albeit to a lesser extent, allowing for slower growth. Substrate induction via AepR^WX^ at the *phnWX* promoter is not essential for growth but is required for efficient utilization of 2AEP. D. CbrAB together with 2AEP is required for complete expression from the *phnWX* promoter. However, 2AEP as the sole C source is sufficient for full expression from the *aepP* promoter. Substrate induction occurs via AepR^WX^ at the *phnWX* promoter, and likely through the homologous AepR^P^ at the *aepP* promoter. In the absence of NtrBC, substrate induction drives expression at both promoters, albeit to a lesser extent, allowing for slower growth. Substrate induction via AepR^WX^ at the *phnWX* promoter is not essential for growth but is required for efficient utilization of 2AEP. N.B. arrows do not necessarily indicate direct interactions between regulators and promoter regions.

This study also demonstrated an important, but non‐essential, role for the LysR‐like AepR whose gene is located adjacent to the *phnWX* operon in BIRD‐1. Both growth and β‐galactosidase activity from the *phnWX* promoter is severely curtailed in the *aepR* ^*WX*^ mutant, and is only detectable above background levels when 2AEP is present as the sole C source. This low‐level expression is consistent with our previous proteomics data, which revealed AepX, PhnW and PhnX are synthesized at low levels in Pi‐limited cells (Lidbury *et al*., 2016). As such AepR^WX^, alongside the other AepR homologues possessed by BIRD‐1 (AepR^XVW^ and AepR^P^) (Murphy *et al*., 2021), provide candidates for substrate‐induction regulators. We speculate that each AepR regulator interacts only with its adjacent operon, given that neither could compensate for AepR^WX^. We therefore present a model of aminoethylphosphonate regulation in BIRD‐1 in Fig. 7. Our finding that nutrient limitation in the absence of 2AEP was insufficient to drive complete expression from either the *aepP* or *phnWX* promoter regions in BIRD‐1 agrees with the N limitation proteomics data we present here and our previous proteomics data (Murphy *et al*., 2021), that shows the presence of 2AEP in addition to N limitation is required to detect synthesis of PhnWX. Whilst our previous proteomics data did detect low levels of PhnWX and AepXVW synthesis in response to P limitation in BIRD‐1, we did not detect these in *Pseudomonas fluorescens* SBW25 (Lidbury *et al*., 2016). Collectively, our combined data demonstrate a role for substrate inducible expression, controlled by AepR, in combination with nutrient limitation, controlled by either PhoBR, NtrBC, or CbrAB.

Given that β‐galactosidase activity from all aminophosphonate promoters is reduced in a Δ*phnWX* strain that is incapable of 2AEP catabolism (Murphy *et al*., 2021), it is likely that a breakdown product of 2AEP is (at least partially) responsible for substrate induction via AepR proteins. We hypothesize that phosphonoacetaldehyde is the most likely candidate molecule, as, like AepR homologues (Murphy *et al*., 2021), this intermediate is common to both PhnWX and PhnWAY pathways of 2AEP breakdown (Villarreal‐Chiu *et al*., 2012), as well as the pathway of 1‐OH‐2AEP degradation by PbfA (Zangelmi *et al*., 2021). Indeed, the gene (*pbfA*) encoding PbfA is located in both *phnWX* and *phnWAY* operons (Zangelmi *et al*., 2021). Phosphonoacetaldehyde is only produced during phosphonate degradation, unlike the final breakdown products alanine, acetaldehyde/acetate and phosphate.

2AEP can act as a replacement headgroup for classical phospholipids (Mukhamedova and Glushenkova, 2000; Kafarski, 2019) and can also form part of bacterial polysaccharides (Baumann *et al*., 1992; Vinogradov *et al*., 2001). However, the source of 2AEP in the rhizosphere is unclear. Ecologically important soil protists (Gao *et al*., 2019; Xiong *et al*., 2020) produce abundant phosphonolipids (Mukhamedova and Glushenkova, 2000; Kafarski, 2019). Plants also synthesize phosphonates (Wieczorek *et al*., 2021), though the importance of this to the rhizosphere has yet to be determined. Additionally, genomic evidence suggests soil bacteria are also a major source of 2AEP (Li and Horsman, 2022). Moreover, the abundance of 2AEP transporters and degradation pathways in bacterial meta‐omics datasets suggests 2AEP production is ubiquitous (Murphy *et al*., 2021). We speculate that 2AEP acquisition provides a clear advantage during nutrient limiting growth conditions which are frequently observed in plant rhizospheres (Bell *et al*., 2014; Cui *et al*., 2018), by expanding the metabolic repertoire of BIRD‐1 to utilize substrates associated with the plant microbiome (Kuramae *et al*., 2020; Akinola *et al*., 2021). Indeed, 2AEP catabolism may present a key nutrient driving plant–*Pseudomonas* interactions and partially explain why this genus forms abundant components of rhizosphere, rhizoplane and root endophyte communities (Robinson *et al*., 2016; Rathore *et al*., 2017). Importantly, utilization of 2AEP as sole N, or C and N source (Ternan and Quinn, 1998; Murphy *et al*., 2021) releases bioavailable Pi into the surrounding environment, which may be an important process in recycling Pi in the rhizosphere.

The putative Ntr regulon elucidated by our proteomics dataset has much in common with previous transcriptomics datasets retrieved from *P*. *putida* KT2440 (Hervás *et al*., 2008) and *P*. *aeruginosa* PA14 (Alford *et al*., 2020). Both strains possess homologues of *aepP* and *phnWX*, neither of which was differentially transcribed in either strain (Hervás *et al*., 2008; Alford *et al*., 2020). In agreement with Hervás *et al*. (2009) we found that the AmtB ammonium channel and the sole pseudomonad PII protein GlnK, which are transcribed from a single operon, were significantly downregulated in the *ntrBC* mutant under low N conditions. However, it was also significantly downregulated under high N conditions in the *ntrBC* mutant. To our knowledge, this is the first time this has been demonstrated. Indeed, examination of the raw dataset showed neither AmtB nor GlnK was detected in the *ntrBC* mutant under either high or low N conditions. This is in keeping with the suggestion by Hervás *et al*. that *glnK* and *amtB* expression may be sensitive to low concentrations of phosphorylated NtrC, and high levels of GlnK, induced by NtrBC, serve to quickly deactivate Ntr regulon activity if sufficient N is obtained (Hervás *et al*., 2009). The absence of transaminase (PhnW) and phosphonatase (PhnX) from this dataset is interesting given that NtrBC is required for proper growth on 2AEP under N limited conditions. By conducting β‐galactosidase assays we discovered that the presence of 2AEP is required, and that global omics approaches can miss regulon members which require substrate induction. Differences in β‐galactosidase expression in the Δ*ntrBC* and Δ*cbrAB* mutants compared to wild‐type are small compared to what would be expected given the differences in growth in these strains, suggesting a discrepancy between protein activity and gene transcription. The mechanism behind this discrepancy is unclear. However, the involvement of post‐transcriptional mechanisms of regulation, such as catabolite repression (Moreno *et al*., 2012; Moreno *et al*., 2015), cannot be ruled out, especially given the interaction between CbrAB and catabolite repression (García‐Mauriño *et al*., 2013). Equally, it remains to be established whether RNA chaperones such as Crc/Hfq (Moreno *et al*., 2015) interact with mRNAs in the BIRD‐1 phosphonate operons.

In summary, we demonstrate that BIRD‐1 requires the two‐component master regulators of nutrient stress PhoBR, NtrBC and CbrAB to optimally use 2AEP as the sole P, N, or C source respectively. However, the presence of 2AEP, as well as an as‐yet‐unidentified metabolite, is required for complete expression of the phosphonate operons and maximal growth. Through mutagenesis of AepR^WX^, we also identified a role for the LysR‐like regulators found adjacent to aminoethylphosphonate operons in bacteria, driving substrate induction at their adjacent operons. Thus, BIRD‐1 employs a twofold regulatory strategy for 2AEP transport and catabolism comprising nutrient stress responses via PhoBR, NtrBC, or CbrAB, as well as substrate induction via AepR.

## Experimental procedures

### Bacterial strains and growth conditions

*P. putida* BIRD‐1 was maintained on Luria–Bertani (LB) agar plates (1.5% wt./vol.) at 30°C. Mutants were also maintained on LB agar plates but with the addition of the appropriate antibiotic (see below). For all growth curves and proteomics experiments, *P*. *putida* BIRD‐1 strains were grown in modified minimal media A (Lidbury *et al*., 2016) using 20 mM sodium succinate as the sole C source or 1.5 mM KH_2_PO_4_ as the sole P source, where applicable. Under high N conditions, 5.6 mM NH_4_Cl was used as the sole nitrogen source, whilst under low N conditions 1.5 mM NH_4_Cl was used. 2AEP was added at a final concentration of 1.5 mM when used as sole N or sole P source, and at 7.5 mM when used as the sole C source. Culture experiments were either performed in a FLUOstar Omega 96‐well plate reader using Sarstedt 96‐well plates, incubated at 30°C with 200 rpm shaking, or in 100 ml flasks incubated at 30°C with 180 rpm shaking.

### Generation of mutants and *lacZ* expression strains

Mutants were generated according to the protocol detailed in Lidbury *et al.* (*2016*) and Murphy *et al*. (2021). Briefly, regions of genomic DNA corresponding to the ends of the genes targeted for knockout were cloned into pk18*mobsacB* together with a gentamicin resistance cassette using the HiFi DNA assembly kit (New England Biolabs). *Escherichia coli* S17.1 λ pir cells were transformed by electroporation and used to mobilize plasmids by conjugation (18 h at 30°C). Transconjugants were selected with gentamicin (50 μg ml^−1^), using chloramphenicol (10 μg ml^−1^) as counter selection. Single crossovers were confirmed by PCR and double crossovers were selected by plating on LB with gentamicin and 10% wt./vol. sucrose, with the exception of the Δ*cbrAB* mutants where minimal media A plates with gentamicin and 10% wt./vol. sucrose were used. Homologous recombinants were confirmed using PCR and Sanger sequencing. Promoter fusion plasmids were created using pBIO1878 (Todd *et al*., 2012). Briefly, promoter regions (250 bp prior to, but not including, the translation start site) were amplified using PCR primers containing restriction sites, subcloned into pGEM‐T easy vectors (Promega), purified by restriction digest and gel purification, and ligated into restriction digested pBIO1878 using T4 DNA ligase (Promega). Again, plasmids were electroporated into *E*. *coli* S17.1 λ pir, and mobilized into *P*. *putida* strains via conjugation. Transconjugants were selected on LB containing spectinomycin (50 μg ml^−1^) and tetracycline (20 μg ml^−1^), using chloramphenicol (10 μg ml^−1^) as counter selection, prior to PCR confirmation. A full list of primers and plasmids used in this study is presented in Supplementary Table 1.

### Proteomics sample preparation and experimental analysis

To identify differentially synthesized proteins in wild‐type versus Δ*ntrBC* strains under high and low N conditions (*n* = 3), total protein was collected by pelleting cells after overnight growth. Prior to this, to account for differences in growth yield between high and low N conditions, OD_600_ readings were taken and volumes were adjusted so approximately equal cells were sampled for each condition. Cells were re‐suspended in 1 ml Tris–HCl (20 mM, pH 7.5) and disrupted by sonication. Protein concentrations were determined using a Bradford assay and equivalent quantities (~20 μg) of each sample were loaded into lithium dodecyl sulfate buffer (Abcam) onto a 4%–20% Bis‐Tris sodium dodecyl sulfate (SDS) precast gel (Abcam) for a short (~1 cm) run. Following Coomassie Blue staining to ensure protein had been loaded correctly, gel slices were destained using 50% wt./vol. ethanol in 50 mM ammonium bicarbonate, dehydrated with 100% ethanol, reduced and alkylated with Tris‐2‐carboxyethylphosphine and iodoacetamide, washed again with 50% wt./vol. ethanol in 50 mM ammonium bicarbonate, dehydrated in 100% ethanol and digested overnight with trypsin. Peptides were extracted using 25% acetonitrile 5% formic acid, vacuum dried and re‐suspended in 2.5% acetonitrile 0.05% trifluoroacetic acid and analysed using an Orbitrap Fusion Ultimate 3000 RSLCNano system (Thermo Scientific) in electrospray ionization mode at the University of Warwick Proteomics Research Technology Platform.

Tandem mass spectrometry (MS/MS) files were searched against the *P*. *putida* protein sequence database (NC_017530.1) using MaxQuant (Tyanova *et al*., 2016a) with default settings. Label‐free quantification (LFQ) was used for quantification. As done previously (Murphy *et al*., 2021) Perseus 1.6.15.0 (Tyanova *et al*., 2016b) was used to analyse the data and identify differentially synthesized proteins using Log_2_ LFQ values and *t*‐tests using FDR adjusted *p*‐values. Proteins absent from more than two replicates in any condition were discarded, whilst remaining missing values were replaced with inputs from a normal distribution using default parameters. This dataset is provided as a supplementary.

### β‐Galactosidase assays

Assays were performed as described by Miller (1972). Briefly, cultures were incubated with replete nutrients overnight, prior to centrifugation and re‐suspension in fresh media (performed twice). Once the requisite nutrients for each condition were added, cultures were then incubated at 30°C for 5 h with shaking at 180 rpm, at which point the OD_600_ was measured. Cells were then twice centrifuged and re‐suspended in Z buffer (8.54 g L^−1^ Na_2_HPO_4_, 5.5 g L^−1^ NaH_2_PO_4_·H_2_O, 0.75 g L^−1^ KCl, 0.25 g L^−1^ MgSO_4_·7H_2_O, pH 7.0). Two drops of chloroform and one drop of 0.1% wt./vol. SDS were added to 1 ml sample, and samples vortexed briefly to permeabilize the cells. Samples were briefly pre‐incubated at 30°C and 200 μl of 4 g L^−1^ O‐nitrophenyl‐β‐d‐galactopyranoside was added. Samples were incubated until colour became apparent, with time recorded. Reactions were stopped with 500 μl 1 M Na_2_CO_3_, and samples were incubated at 30°C for 5 min, before centrifugation to remove cell debris. OD_420_ was measured and β‐galactosidase activity was calculated as Miller units according to the formula activity = (OD_420_ × 1000)/(OD_600_ × time × volume).

## Supporting information


**Appendix** **S1**: Supporting Information.
