# Supplementary material for: 2‐Aminoethylphosphonate utilization in Pseudomonas putida BIRD‐1 is controlled by multiple master regulators
Source: Environ Microbiol. 2022 Mar 8;24(4):1902–17. doi: 10.1111/1462-2920.15959 (PMC9311074; doi:10.1111/1462-2920.15959)
Supplement: Supplementary file 1 — Appendix S1: Supporting Information. [file EMI-24-1902-s001.docx]

**Supplementary information**

**2-aminoethylphosphonate utilisation in *Pseudomonas putida* BIRD-1 is controlled by multiple master regulators**

Andrew R. J. Murphy^1^, David J. Scanlan^1^, Yin Chen^1^, Gary D. Bending^1^, John P. Hammond^2^, Elizabeth M. H. Wellington^1^, Ian D.E.A. Lidbury^3^*

^1^School of Life Sciences, University of Warwick, Gibbet Hill Road, Coventry, UK

^2^School of Agriculture, Policy, and Development, University of Reading, Earley Gate, Whiteknights, Reading, UK

^3^Plants, Photosynthesis and Soil Research Cluster, School of Biosciences, University of Sheffield, Sheffield, UK

Corresponding author: [i.lidbury@sheffield.ac.uk](mailto:i.lidbury@sheffield.ac.uk)

Address:

Dr. Ian Lidbury

Plant, Photosynthesis & Soil research cluster

School of Biosciences

University of Sheffield

Sheffield, UK

Tel: 0114 222 0137 (Office)

**Running Title**

Dual regulatory mechanisms for 2AEP utilisation

Table S1. Primers and plasmids used in this study

| Primer | Sequence | Plasmid | Used For |
| --- | --- | --- | --- |
| NtrBC ArmA_fwd | TACGAATTCGAGCTCGGTACCCGGG  CGATTGAGCGCAAATCCAG | pkmob*sacB*-*ntrBC* | Cloning region A of *ntrBC* |
| NtrBC ArmA_rev | CTCTAGAGTCGACTGGCCTTGATGC  AGTATC | pkmob*sacB*-*ntrBC* | Cloning region A of *ntrBC* |
| NtrBC_Gent_fwd | TGCATCAAGGCCAGTCGACTCTAGA  GGATCCCCGG | pkmob*sacB*-*ntrBC* | Cloning of *gm* cassette for *ntrBC* knockout |
| NtrBC_Gent_rev | TTCAGCAGTTCTGTTGGCCGCGGCG  TTGTGA | pkmob*sacB*-*ntrBC* | Cloning of *gm* cassette for *ntrBC* knockout |
| NtrBC ArmB_fwd | ACGCCGCGGCCAACAGAACTGCTGA  ACCTGC | pkmob*sacB*-*ntrBC* | Cloning region B of *ntrBC* |
| NtrBC ArmB_rev | CGACGGCCAGTGCCAAGCTTGCATG  GGGGATTTGTGCCTTCGAG | pkmob*sacB*-*ntrBC* | Cloning region B of *ntrBC* |
| CbrAB ArmA_fwd | ATTCGAGCTCGGTACCCGGGGCTTT  ACCTGTAGGAGCG | pkmob*sacB*-c*brAB* | Cloning region A of *cbrAB* |
| CbrAB ArmA_rev | TAGAGTCGACTGTAGGTAAGTGGGT  GGC | pkmob*sacB*-c*brAB* | Cloning region A of *cbrAB* |
| CbrAB_Gent_fwd | CTTACCTACAGTCGACTCTAGAGGA  TCCCCGG | pkmob*sacB*-c*brAB* | Cloning of *gm* cassette for *cbrAB* knockout |
| CbrAB_Gent_rev | AGGTCGCTCATGGCCGCGGCGTTGT  GAC | pkmob*sacB*-c*brAB* | Cloning of *gm* cassette for *cbrAB* knockout |
| CbrAB_ArmB_fwd | GCCGCGGCCATGAGCGACCTGGAAG  AGG | pkmob*sacB*-c*brAB* | Cloning region B of *cbrAB* |
| CbrAB ArmB_rev | GCCAGTGCCAAGCTTGCATGAATGG  GTTGAGCGGGTTC | pkmob*sacB*-c*brAB* | Cloning region B of *cbrAB* |
| AepXVW_fwd_KpnI | TAAGCAGGTACCGGCCGGTTCGCAT  TCTAGA | pBIO-*aepXVW*-pr | Cloning of *aepXVW* promoter |
| AepXVW_rev_PstI | TAAGCACTGCAGGGCATGGCCTCAT  CGAAGAG | pBIO-*aepXVW*-pr | Cloning of *aepXVW* promoter |
| AepP_fwd_KpnI | TAAGCAGGTACCCCGACATGACAAC  CTCAGTAAC | pBIO-*aepP*-pr | Cloning of *aepP* promoter |
| AepP_rev_SphI | TGGCATGCATGCGTAAGTCTGACCT  CTTGCTCA | pBIO-*aepP*-pr | Cloning of *aepP* promoter |
| PhnWX_fwd_KpnI | TAAGCAGGTACCGGTGATCTGGTAG  TGCTCC | pBIO-*phnWX*-pr | Cloning of *phnWX* promoter |
| PhnWX_rev_SphI | TGGCATGCATGCTCCGTTATTCCTC  ACAAGCGG | pBIO-*phnWX*-pr | Cloning of *phnWX* promoter |
| AepR^WX^_ArmA_fwd | attcgagctcggtacccgggcttcg  ccaggcgttggccg | pkmob*sacB*-*aepR^WX^* | Cloning region A of *aepR^WX^* |
| AepR^WX^_ArmA_rev | gccctcgccgcggtcaccgccggct  ggc | pkmob*sacB*-*aepR^WX^* | Cloning region A of *aepR^WX^* |
| AepR^WX^_ArmB_fwd | gcggtgaccgcggcgagggccttgt  caa | pkmob*sacB*-*aepR^WX^* | Cloning region B of *aepR^WX^* |
| AepR^WX^_ArmB_rev | taaaacgacggccagtgccaaattc  gtcgttgcagcgc | pkmob*sacB*-*aepR^WX^* | Cloning region B of *aepR^WX^* |


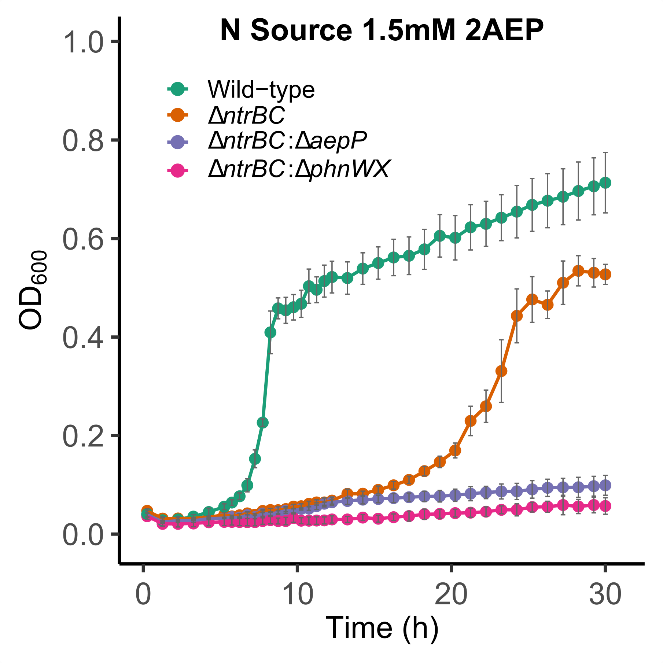


Supplementary Figure 1. 2AEP utilisation by *P. putida* BIRD-1 as the sole N source. Growth (*n* = 4) of *P. putida* BIRD-1 wild-type, Δ*ntrBC*, Δ*ntrBC*:Δ*aepP*, and Δ*ntrBC*:Δ*phnWX* on 2AEP as the sole N source. Error bars denote the standard deviation of the mean.


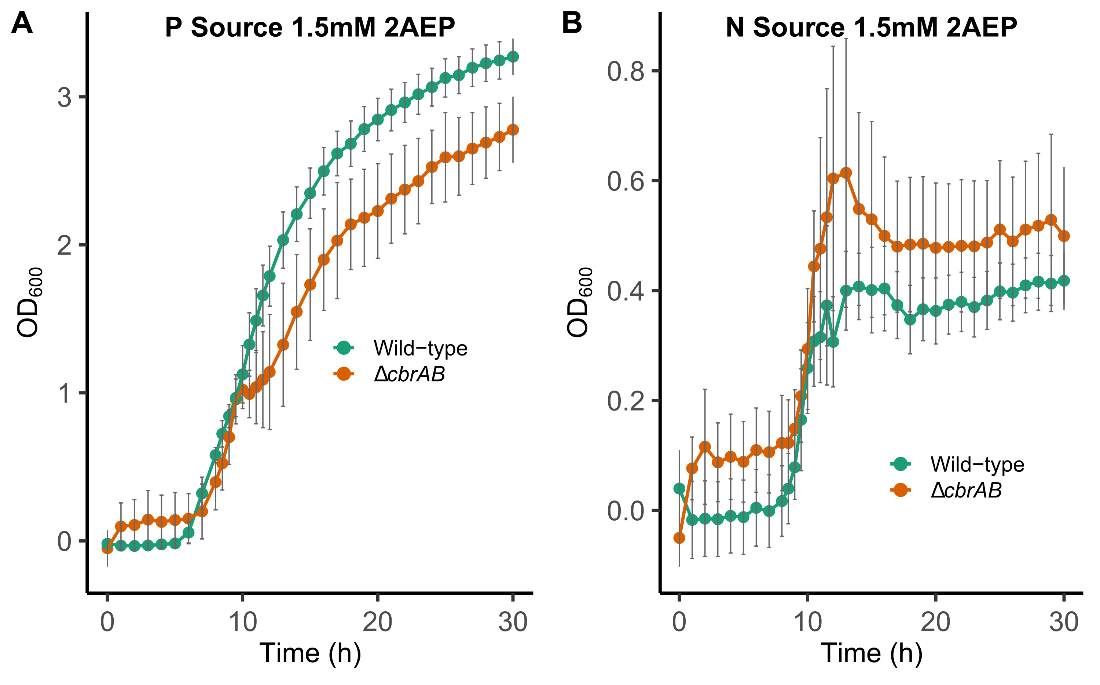


Supplementary Figure 2. 2AEP utilisation by *P. putida* BIRD-1 Δ*cbrAB*. Growth (*n* = 4) of *P. putida* BIRD-1 wild-type and Δ*cbrAB* on 2AEP as the sole P source (**A**) or sole N source (**B**). Error bars denote the standard deviation of the mean.


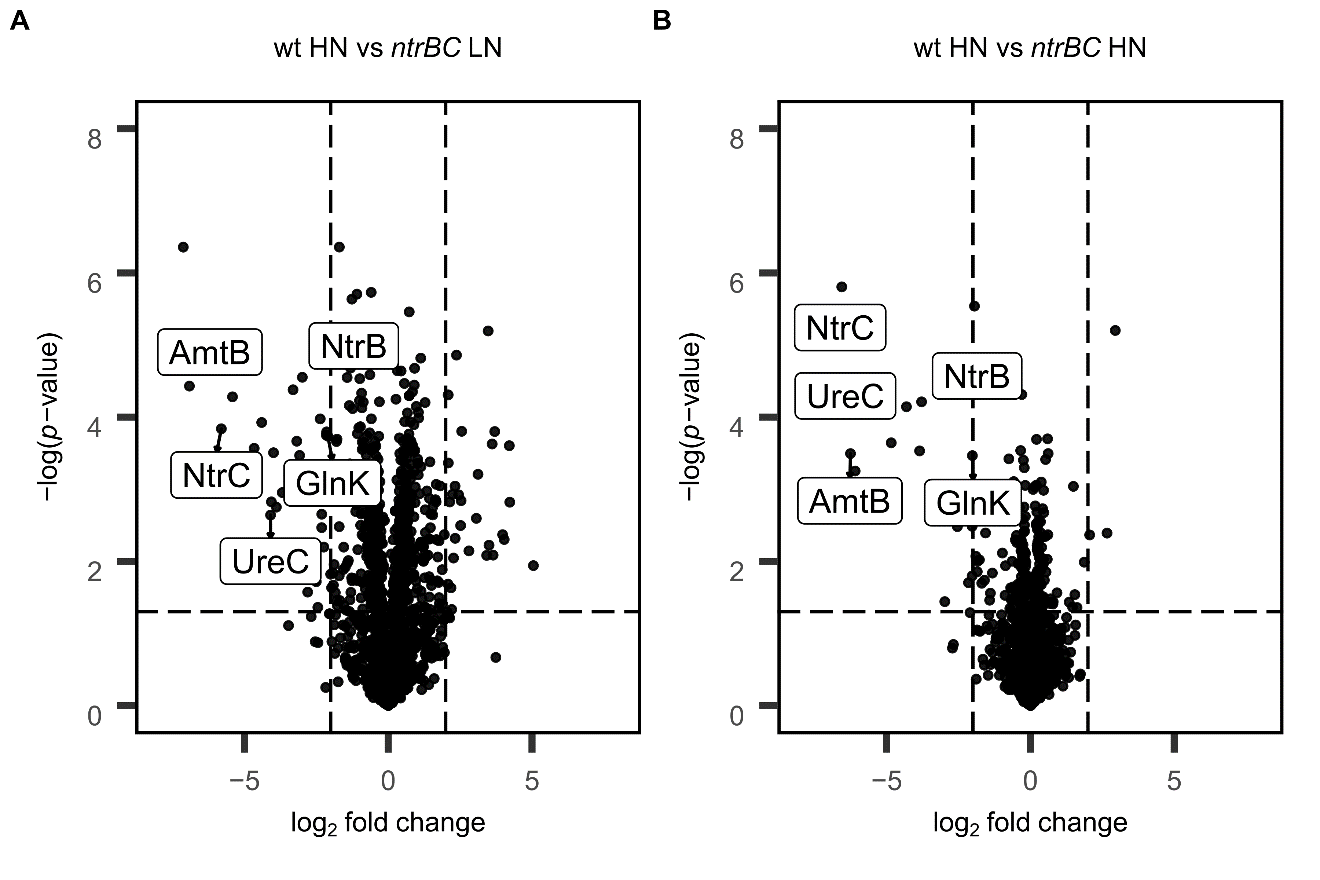


Supplementary Figure 3. Whole-cell protein profiles (*n* = 3) for *P. putida* BIRD-1 grown under either High N (HN – 5.6mM NH_4_) or Low N (LN – 1.5mM NH_4_) conditions. Wild-type HN vs Δ*ntrBC* LN (**A**), wild-type HN vs Δ*ntrBC* HN (**B**). Log_2_ fold change represents the difference in mean Log_2_ Label Free Quantification (LFQ) values between each treatment. The statistical value on the y-axis is generated from Q values (FDR corrected P values). Vertical dashed lines represent a Log_2_ LFQ difference > −2 or <2. The horizontal dashed line illustrates a cut-off for a significant -Log_10_(*p*-value) (p < 0.05). Proteins significantly down-regulated in the Δ*ntrBC* mutant in both HN and LN conditions are labelled.


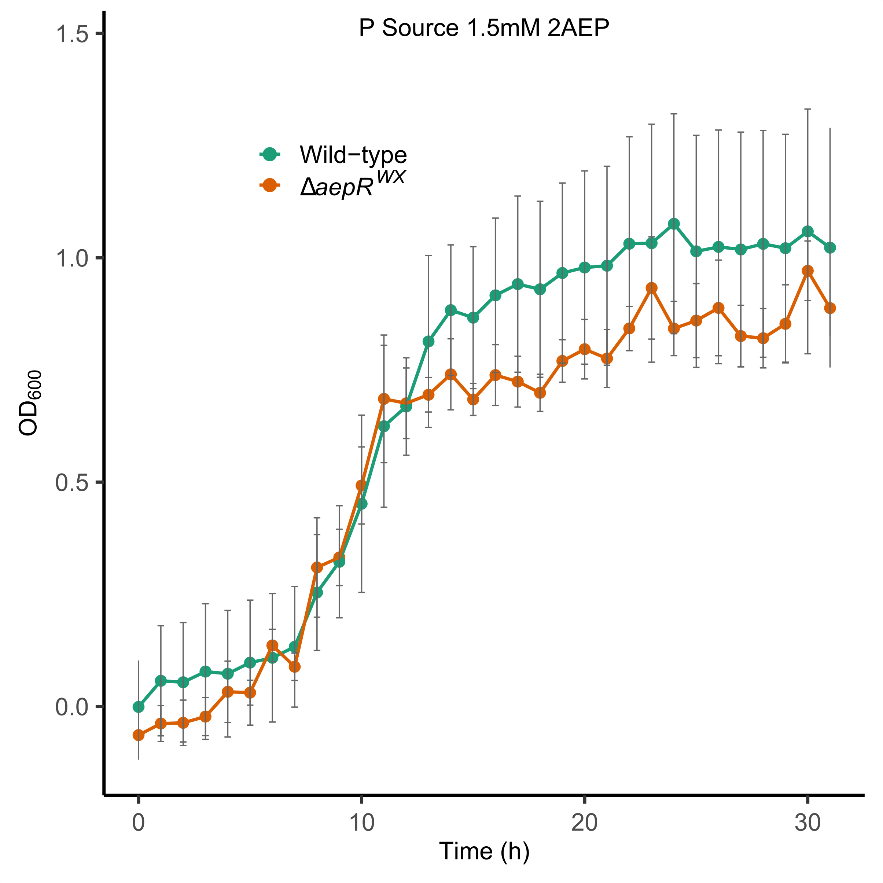


Supplementary Figure 4. 2AEP utilisation by *P. putida* BIRD-1 Δ*aepR^WX^*. Growth (*n* = 4) of *P. putida* BIRD-1 wild-type and Δ*aepR^WX^* on 2AEP as the sole P source (**A**) (1.5 mM). Error bars denote the standard deviation of the mean.
